# Peroxynitrite/PKR Axis Modulates the NLRP3 Inflammasome of Cardiac Fibroblasts

**DOI:** 10.3389/fimmu.2020.558712

**Published:** 2020-09-25

**Authors:** Ting Lan, Aibin Tao, Xuemei Xu, Peter Kvietys, Tao Rui

**Affiliations:** ^1^Division of Cardiology, Department of Medicine, The Affiliated People's Hospital of Jiangsu University, Zhenjiang, China; ^2^Critical Illness Research, Lawson Health Research Institute, London, ON, Canada; ^3^Department of Laboratory Medicine, The Affiliated Hospital of Xuzhou Medical University, Xuzhou, China; ^4^Critical Care Western, Schulich School of Medicine and Dentistry, Western University, London, ON, Canada; ^5^Department of Physiological Sciences, College of Medicine, Alfaisal University, Riyadh, Saudi Arabia; ^6^Departments of Medicine, Pathology and Laboratory Medicine, Schulich School of Medicine and Dentistry, Western University, London, ON, Canada

**Keywords:** NLRP3 inflammasome, cardiac fibroblast, protein kinase R, peroxynitrite, IL-1β, caspase 1, LPS/ATP

## Abstract

Sepsis/endotoxemia activates the NLRP3 inflammasome of macrophages leading to the maturation and release of IL-1β, an important mediator of the inflammatory response. Reactive oxygen species have been implicated in NLRP3 inflammasome activation. Further, our preliminary studies indicated that LPS challenge of cardiac fibroblasts could phosphorylate protein kinase R (PKR) on threonine 451 and increase message for pro-IL-1 β. Thus, the major aim of the present study was to address the role of PKR and the oxidant, peroxynitrite, in the two-tiered function of the NLRP3 inflammasome (priming and activation).

**Materials and Methods:** Isolated murine fibroblasts were primed with LPS (1 μg/ml) for 6 h and subsequently activated by an ATP (3 mM) challenge for 30 min to induce optimum functioning of the inflammasome. Increased levels of NLRP3 and pro-IL-1β protein (Western) were used as readouts for inflammasome priming, while activation of caspase 1 (p20) (Western) and secretion of IL-1β (ELISA) were indicative of inflammasome activation.

**Results:** Inhibition of PKR (PKR inhibitor or siRNA) prior to priming with LPS prevented the LPS-induced increase in NLRP3 and pro-IL-1β expression. Further, inhibition of PKR after priming, but before activation, did not affect NLRP3 or pro-IL-1β protein levels, but markedly reduced the activation of caspase 1 and secretion of mature IL-1β. In a similar fashion, a peroxynitrite decomposition catalyst (Fe-TPPS) prevented both the priming and activation of the NLRP3 inflammasome. Finally, pretreatment of the fibroblasts with Fe-TPPS prevented the LPS-induced PKR phosphorylation (T451).

**Conclusion:** Our results indicate that peroxynitrite-/PKR pathway modulates priming and activation of NLRP3 inflammasome in LPS/ATP challenged cardiac fibroblasts.

## Introduction

Sepsis is an exaggerated systemic inflammatory response to infection that significantly contributes to the high mortality of patients in intensive care units (ICUs) (1, 2). Septic patients are characterized by hypotension, ischemia, and multiple organ failure, all of which can be fatal (3). Among the various organ systems that fail in sepsis, the heart is one of the most frequently affected. Indeed, myocardial dysfunction is a crucial element in the pathogenesis of multiple organ failure in septic patients (4). While the precise mechanism(s) that lead to myocardial dysfunction is not clear, it is generally accepted that the excessive generation of cytokines, colloquially referred to as “cytokine storm,” plays a pivotal role (5).

One of the major cytokines implicated in the cytokine cascade of sepsis is IL-1β (5, 6). IL-1β is generated upon activation of membrane bound or cytosolic pattern recognition receptors (PRRs), which detect pathogen-associated molecular patterns (PAMPS) or damage-associated molecular patterns (DAMPs). A prominent intracellular PRR in sepsis is the NLRP3 (NOD-like, leucine rich repeat domains, and pyrin domain-containing protein 3) (7, 8). In quiescent cells, NLRP3 is present as a latent monomer. Upon appropriate stimulation, NLRP3 recruits ASC (an adapter protein) and pro-caspase 1, eventually forming a functional NLRP3 inflammasome complex by oligomerization (7–10). In general, a two-step process is involved in the optimal functioning of the NLRP3 inflammasome: priming and subsequent activation (7). In this scenario, a priming signal induced by extracellular PAMPs (e.g., LPS) will activate the TLR4/NFκB pathway to increase synthesis of NLRP3 and pro-IL-1β. Subsequently, a second extracellular signal by DAMPs (e.g., ATP) will induce NLRP3 oligomerization with ASC and pro-caspase1. Pro-caspase1 of the assembled complex is converted to the active form (via autocatalysis) and cleaves latent pro-IL-1β to the mature IL-1β, which is subsequently released into the extracellular space. A feed-forward mechanism for IL-1β generation exists, i.e., IL-1β can activate the NLRP3 inflammasome and vice versa (7, 8, 11), representing a potential mechanism of generating the exaggerated cytokine response in sepsis.

Existing data implicate a diverse array of intracellular mediators operating downstream of plasma membrane PRRs to activate the intracellular NLRP3 inflammasome (7, 12, 13). Many of the proposed mediators are involved in cellular oxidative/nitrosative stress. However, substantial controversy exists in the field and no clear consensus has been reached (7). For example, reactive oxygen species (ROS) can be involved (1) in the priming step (8, 14), (2) in the activation step (7), or (3) both priming and activation steps of NLRP3 inflammasome regulation (12, 13, 15). The major sources of ROS, primarily superoxide, are NADPH oxidase and mitochondria (6, 7, 15, 16). The evidence supporting a role for nitric oxide (NO) in the regulation of NLRP3 inflammasome is rather scant and controversial. NO generated by inducible NOS (iNOS) inhibits LPS-induced priming of NLRP3 inflammasome (17), while NO generated during ATP-induced activation of the inflammasome promotes processing of IL-1β (18). In the latter study, the role of NO was to serve as a co-reactant with NADPH oxidase-derived superoxide to form peroxynitrite (18). However, the role of peroxynitrite in activation of the NLRP3 inflammasome in macrophages remains controversial (19, 20). Of note, peroxynitrite plays a role in sepsis-induced cardiomyopathy in a variety of animal models, as well as humans (21–23).

Protein kinase R (PKR) is a serine/threonine kinase containing a N-terminal dsRNA-binding domain and a C-terminal kinase domain (24). It is an anti-viral protein by virtue of its ability to bind dsRNA and inhibit protein translation (25). Of relevance herein, PKR is also a sensor and responder to intracellular stress and mediator of innate immunity (24, 25). Indeed, current opinion regards PKR as an important mediator of NLRP3 inflammasome assembly and activation (12, 26, 27). However, this view is not without distractors. For example, activation of PKR can promote (28–31), suppress (32), or have no effect (33) on NLRP3 inflammasome generation of mature IL-1β. Interestingly, mice can be rescued from the lethal effects of endotoxemia or sepsis by pharmacologic inhibition of PKR (34).

The bulk of the information available on the regulation of NLRP3 inflammasome priming and activation is derived from studies using monocyte/macrophage cells, presumably due to the role of this inflammasome in the innate immune response. However, a functional tripartite NLRP3 inflammasome is also present in non-immune cells of the heart, specifically, cardiomyocytes (35) and cardiac fibroblasts (36, 37). We have previously shown that peritonitis or systemic LPS results in elevations of myocardial IL-1β, myocardial dysfunction, and, importantly, death; effects ameliorated by inhibition of the NLRP3 inflammasome (38). Further, the NLRP3 inflammasome activity of cardiac fibroblasts generates IL-1β, which subsequently impairs the function of cardiac myocytes and induces apoptosis (38, 39). In the present study, we have provided evidence to support a role for a peroxynitrite/PKR pathway in the regulation of NLRP3 inflammasome pathway in cardiac fibroblasts with relevance to endotoxemia/sepsis.

## Materials and Methods

### Cardiac Fibroblast Isolation and Culture

Hearts were excised from adult mice (C57BL/6 background), minced, and digested (Collagenase II, 160 U/ml). After several washing steps, the cell suspension was passed through a nylon mesh (70 μm). Endothelial cells were removed from the filtrate using a magnetic bead technique. The remaining cells were transferred to a humidified incubator (5% CO_2_ at 37°C) for 1 h, after which the adherent cells were mainly fibroblasts; the non-adherent cells (primarily cardiomyocytes) were removed. Finally, the fibroblasts were cultured with Dulbecco's Modified Eagle's Medium (DMEM)-F12 supplemented with 10% fetal calf serum (FCS), 20 mM L-glutamine and 100 U/ml penicillin G, and 100 μg/ml streptomycin. This method yielded a 95% purity of fibroblasts, as determined by their positive staining of a fibroblast marker (ER-TR7) (40). Cells from one to three passages were used for the experiments.

### NLRP3 Inflammasome Priming and Activation

Cardiac fibroblasts were challenged by the well-recognized two-step pathway (7, 8), an initial priming step with LPS (1 μg/ml) for 6 h followed by an activation step with ATP (3 mM) for 30 min (39). After an initial screening procedure (pretreatment followed by LPS/ATP challenges) to identify interventions meriting further study, their potential roles in either priming or activation steps were assessed. To address the priming step, interventions were imposed prior to the initial LPS challenge. Read-outs for the priming step included increases in pro-IL-1β and NLRP3 protein in fibroblasts as assessed by Western blot. Interventions imposed after the LPS challenge, but prior to the ATP challenge, addressed the activation step. Read-outs for the activation step included increases in caspase1 p20 expression (formed during activation of pro-caspase) as assessed by Western blot and release of mature IL-1β into the supernatant as assessed by ELISA.

### Protein Kinase R (PKR)

PKR phosphorylation on an activation threonine site (p-PKR T451) was assessed by Western blot. Loss of function approaches were used to assess a role for PKR; pharmacologic inhibition of PKR, as well as siRNA targeting of PKR. The oxindole-imidazole C16 that targets the ATP-binding site of PKR was used at a concentration of 0.25–0.5 μM. Lipofectamine 2000 (Life Technologies, Burlington, ON, Canada) was used to transfect fibroblasts with 40 nM of either scrambled siRNA or PKR siRNA (sc-36264; Santa Cruz, Dallas, TX, USA). Down regulation of PKR protein expression was confirmed 48 h after the siRNA transfection by Western blot (Supplementary Figure 1). PKR down-regulation was incomplete, a finding consistent with a previous report (41).

### Peroxynitrite

As a peroxynitrite “footprint,” nitrotyrosine levels in fibroblasts were assessed by Western blot. A peroxynitrite decomposition catalyst, 5,10,15,20-Tetrakis (4-sulfonatophenyl porphyrinato iron [III] chloride [Fe-TPPS; CalBiochem]) was used to limit peroxynitrite activity (18).

### Western Blot

Fibroblast levels of relevant proteins were assessed by Western blot as previously described (39). Briefly, fibroblasts were lysed and homogenized with lysis buffer [10 mM Tris (pH 7.4), 150 mM NaCl, 5 mM EDTA, 1% Triton X-100, 10 mM NaF, 1 mM Na_3_VO_4_, 10 mg/mL leupeptin, 10 mg/mL aprotinin, and 20 mM PMSF]. Proteins from each sample were separated on 10–15% sodium dodecyl sulfate-polyacrylamide gels (SDS-PAGE) and transferred to polyvinylidene fluoride (PVDF) membranes. After blocking with 3% bovine serum albumin (Wisent Inc, St-Bruno, QC, Canada), the membranes were probed with primary antibodies against mouse NLRP3, caspase 1 p20 (Adipogen, San Diego, CA, USA) at 1:500 dilution; pro-IL-1β (R and D Systems, Minneapolis, MN, USA) at 1:500 dilution; nitrotyrosine (Santa Cruz, Dallas, TX, USA) at 1:1,000 dilution; and β-actin (Santa Cruz, Dallas, TX, USA) at 1:2,000 dilution. After treatment with appropriate secondary antibodies, the specific bands were detected with an enhanced chemiluminescence (ECL) system and quantified with an imaging densitometer (Bio-Rad Laboratories, Inc., Hercules, CA, USA).

### ELISA

IL-1β in culture supernatants was assessed with a mouse IL-1β ELISA Kit (BD Biosciences, San Diego, CA, USA) according to the manufacturer's instructions. Culture medium was centrifuged at 500 *g* for 5 min, the supernatant was collected, and levels of IL-1β measured (39).

### Statistical Analysis

Each experiment was performed in triplicate. Data are expressed as mean ± SEM and analyzed with SPSS11.0 statistical software. One-way analysis of variance (ANOVA) with Bonferroni's post-test was used to compare data among groups. A *p* < 0.05 was considered as a statistical significance.

## Results

### Preliminary Feasibility Studies

**Figure 1:**
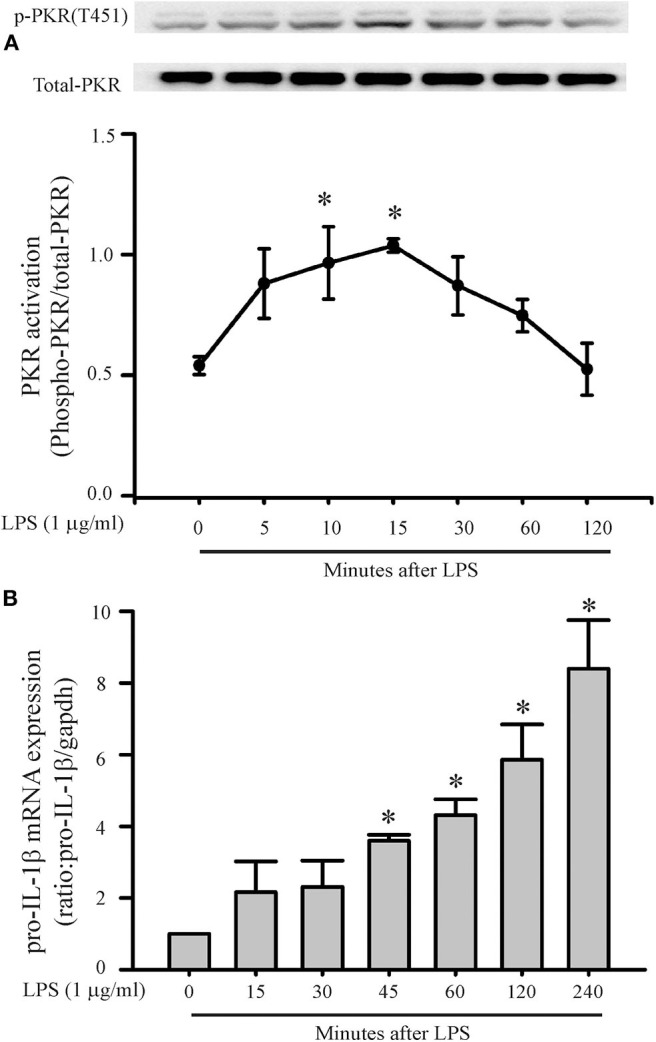
LPS increases PKR phosphorylation and pro-IL-1β mRNA expression. Fibroblasts were challenged with LPS (1 μg/mL) and at indicated times thereafter harvested for assessment of PKR phosphorylation **(A)** or message levels of pro-IL-1β **(B)**. **(A)** Representative Western blot of PKR phosphorylation on the activation threonine 451 (p-PKR [T451]) is shown above and densitometric analysis below. **(B)** Pro-IL-1β levels were assessed by RT-PCR. The forward and reverse primers for Pro-IL-1β were F: *gatccacactctccagctgca*, R: *caaccaacaactgatattctccatg. n* = 3 for both **(A,B)**, *p < 0.05 as compared to control (0 min).

A role for PKR in macrophage NLRP3 inflammasome regulation is controversial (28, 29, 32, 33). Thus, initial feasibility experiments were carried out to determine whether LPS could induce PKR activation and increase pro-IL-1β message in cardiac fibroblasts. Phosphorylation of threonine residues 446 and 451 of PKR are important for kinase activity (26, 42). In the present study, LPS challenge of fibroblasts increased phosphorylation of threonine 451 (T451) of PKR within 10–15 min (Figure 1A). The effect was transient; the phosphorylation status of T451 returned to basal levels within 1–2 h, despite continued LPS exposure. The transient nature of LPS-induced phosphorylation of PKR agrees with previous reports that assessed LPS-induced phosphorylation of PKR in macrophages (43–45).

Message levels of pro-IL-1β in the fibroblasts increased by 45 min of LPS challenge and continued to increase progressively for the duration of study, i.e., 4 h (Figure 1B). Since PKR activation precedes the increase in pro-IL-1β message, PKR may play a role in the LPS-induced priming/activation of the NLRP3 inflammasome in cardiac fibroblasts.

### PKR Regulates NLRP3 Inflammasome Priming/Activation

**Figure 2:**
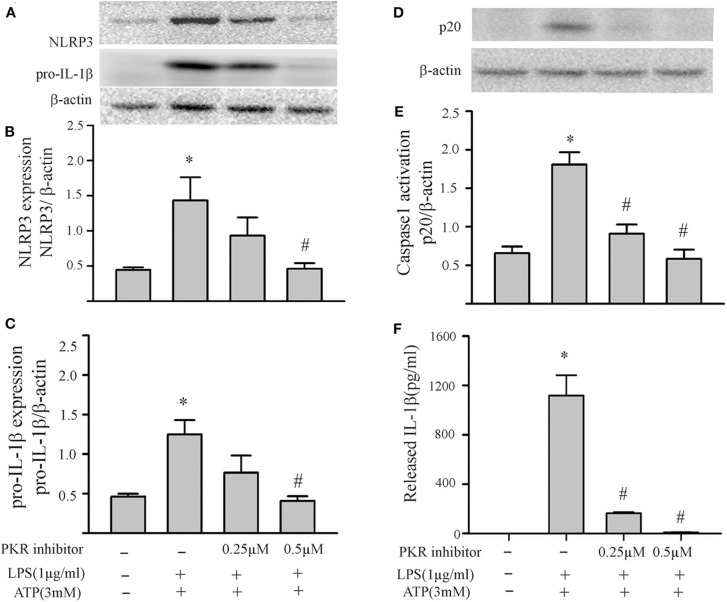
Inhibition of PKR activity decreases NLRP3 inflammasome priming/activation in LPS/ATP challenged fibroblasts. Fibroblasts were pretreated with the PKR inhibitor (C16) for 30 min. Subsequently, the fibroblasts were challenged with LPS (1 μg/mL) for 6 h followed by ATP (3 mM) for 30 min. **(A–C)** NLRP3 and pro-IL-1β protein levels were indices of inflammasome priming. Representative Western blots are shown in **(A)** and densitometric analyses in **(B,C)**. **(D–F)** Caspase 1 activation (p20) and release of mature IL-1β were indices of inflammasome activation. Representative Western blot for caspase 1 p20 is shown in **(D)** and densitometric analysis in **(E)**. Protein levels of IL-1β were assessed in supernatants by ELISA. *n* = 3, *p < 0.05 as compared with control; ^#^*p* < 0.05 as compared with LPS+ATP.

**Figure 3:**
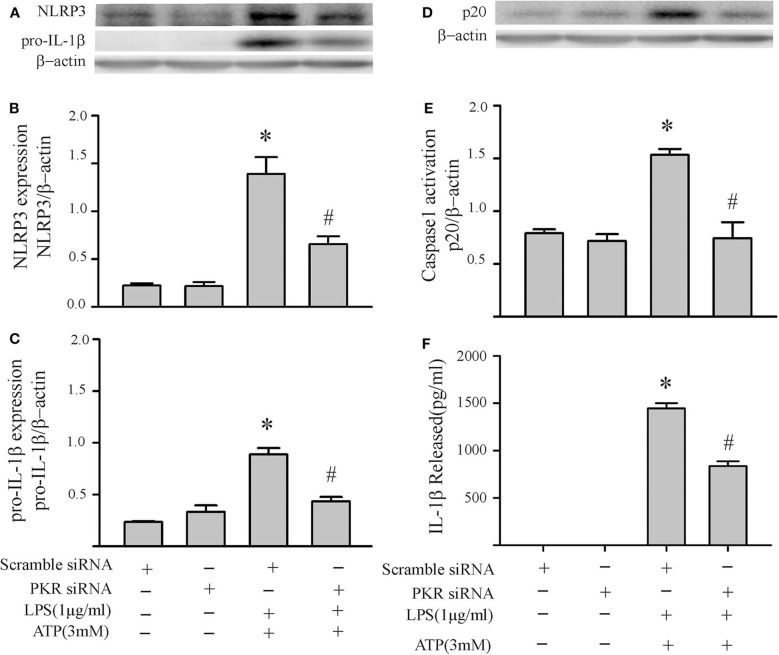
Knock down of PKR by siRNA decreases NLRP3 inflammasome priming/activation in LPS/ATP challenged fibroblasts. Forty-eight hours after transfection with siRNA targeting PKR or scrambled RNA, the fibroblasts were challenged with LPS (1 μg/mL) for 6 h followed by ATP (3 mM) for 30 min. **(A–C)** NLRP3 and pro-IL-1β protein levels were indices of inflammasome priming. Representative Western blots are shown in **(A)** and densitometric analyses in **(B,C)**. **(D–F)** Caspase 1 activation (p20) and release of mature IL-1β were indices of inflammasome activation. Representative Western blot for caspase 1 p20 is shown in **(D)** and densitometric analysis in **(E)**. Protein levels of IL-1β were assessed in supernatants by ELISA. *n* = 3, *p < 0.05 as compared with control; ^#^*p* < 0.05 as compared with LPS+ATP.

The two-step challenge (LPS followed by ATP) increased fibroblast protein levels of NLRP3, pro-IL-1β, caspase1 p-20, as well as release of mature IL-1β into the supernatants (Figure 2). The PKR inhibitor reduced these indices of NLRP3 inflammasome priming and activation. The effects of the PKR inhibitor were dose-dependent, with complete blockade elicited by the highest dose (Figure 2). Qualitatively similar results were obtained by siRNA targeting PKR (Figure 3). However, complete blockade was not uniformly achieved, a predictable outcome considering the incomplete knock-down of PKR (Supplementary Figure 1).

**Figure 4:**
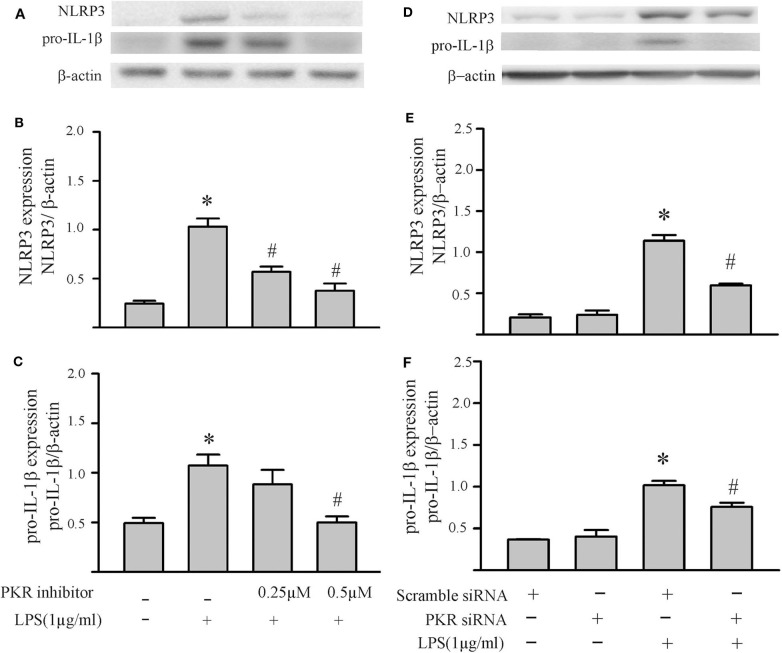
Inhibition of PKR activity or down-regulation of PKR protein decreases LPS-induced priming of the NLRP3 inflammasome. **(A–C)** Fibroblasts were pretreated with the PKR inhibitor for 30 min and subsequently challenged with LPS (1 μg) for 6 h. Representative Western blots are shown in **(A)** and densitometric analyses in **(B,C)**. **(D–F)** Forty-eight hours after transfection with PKR siRNA or scrambled siRNA, the fibroblasts were challenged by LPS (1 μg) for 6 h. Representative Western blots are shown in **(D)** and densitometric analyses in **(E,F)**. *n* = 3, *p < 0.05 as compared with control; ^#^*p* < 0.05 as compared with LPS.

To specifically address the role of PKR in priming the NLRP3 inflammasome of cardiac fibroblasts, the PKR inhibitor was added prior to the LPS challenge and indices of priming assessed thereafter (without further challenge with ATP). Both NLRP3 and pro-IL-1β protein levels were reduced in a dose-dependent manner by the PKR inhibitor (Figures 4A–C). Complete blockade of protein expression was achieved at the highest dose. Qualitatively similar results were obtained with siRNA targeting of PKR (Figures 4D,E). Complete blockade was not achieved, presumably due to the incomplete knock-down of PKR (Supplementary Figure 1).

**Figure 5:**
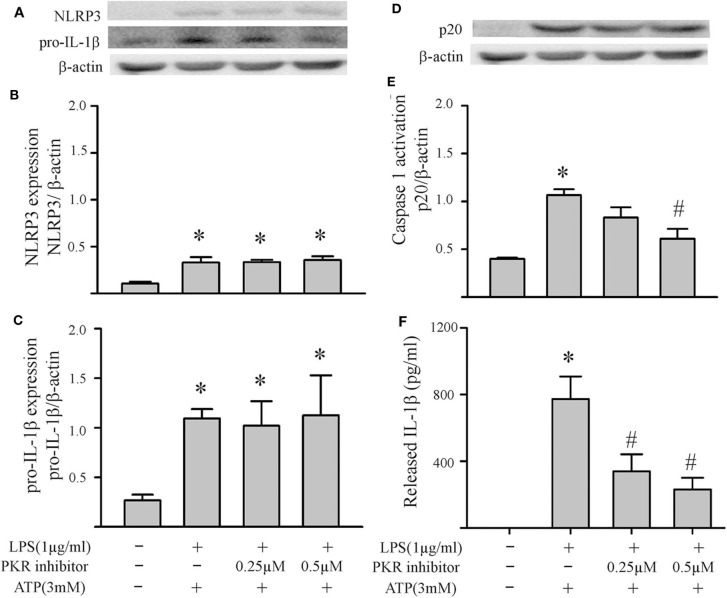
Inhibition of PKR activity decreases ATP-induced activation of the NLRP3 inflammasome. Fibroblasts were challenged with LPS (1 μg/mL) for 6 h. Subsequently, the fibroblasts were treated with the PKR inhibitor C16 for 30 min, followed by ATP (3 mM) for an additional 30 min. **(A–C)** NLRP3 and pro-IL-1β protein levels were indices of inflammasome priming. Representative Western blots are shown in **(A)** and densitometric analyses in **(B,C)**. Of note, NLRP3 and pro-IL-1β were increased after the initial LPS challenge, but the subsequent challenge with ATP was without effect on these two indices of inflammasome priming. **(D–F)** Caspase1 activation (p20) and IL-1β protein levels in the supernatant were indices of inflammasome activation. Representative Western blot for caspase1 p20 is shown in **(D)** and densitometric analysis in **(E)**. Protein levels of IL-1β were assessed in supernatants by ELISA. *n* = 3, *p < 0.05 as compared with control; ^#^*p* < 0.05 as compared with LPS+ATP.

To specifically address the role of PKR in NLRP3 inflammasome activation, the PKR inhibitor was added after the LPS priming step but prior to the ATP activation step. As shown in Figures 5A–C, the PKR inhibitor did not affect the two indices of priming, i.e., LPS-induced increases in NLRP3 and pro-IL-1β protein levels. However, the PKR inhibitor reduced the two indices of inflammasome activation, i.e., increased fibroblast caspase1 p20 protein levels and the amount of mature IL-1β released into the supernatant (Figures 5D,E). Although the effect was dose-dependent, complete blockade of enhanced levels of caspase 1 p20 in the fibroblasts or release of IL-1β was not achieved (compare to Figures 2D,E).

### Peroxynitrite Regulates NLRP3 Inflammasome Priming/Activation

**Figure 6:**
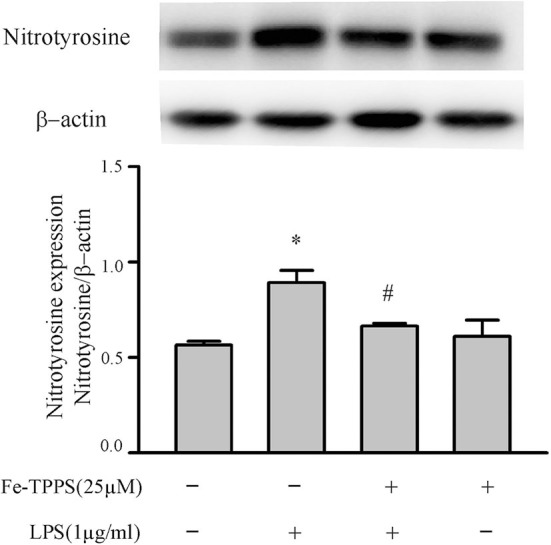
Fibroblast nitrotyrosine levels are increased by a LPS challenge, an effect prevented by a peroxynitrite decomposition catalyst (Fe-TPPS). Fibroblasts were pretreated with Fe-TPPS (25 μM) or vehicle for 30 min and subsequently challenged with LPS for 15 min. Nitrotyrosine levels were assessed by Western blot. Representative blots are shown in upper panel and densitometric analysis in lower panel. *n* = 3, *p < 0.05 as compared with control; ^#^*p* < 0.05 as compared with LPS.

**Figure 7:**
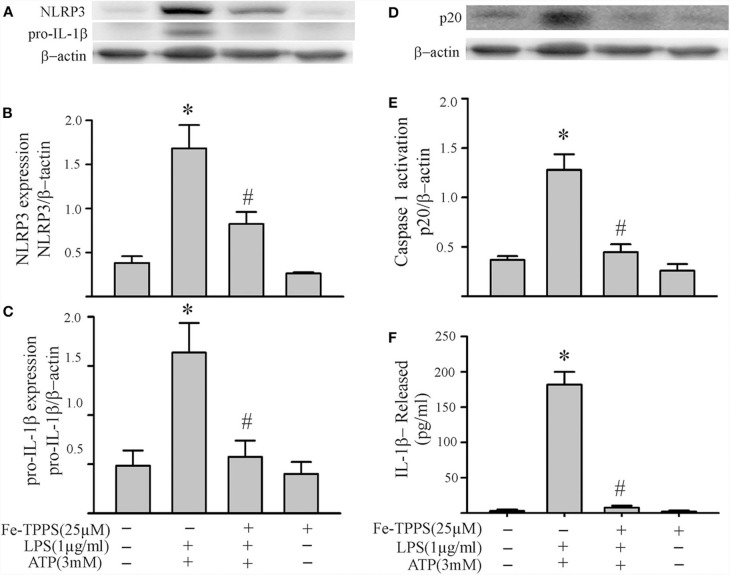
A peroxynitrite decomposition catalyst (Fe-TPPS) decreases NLRP3 inflammasome priming/activation in LPS/ATP challenged fibroblasts. Fibroblasts were pretreated with Fe-TPPS (25 μM) or vehicle for 30 min. Subsequently, they were challenged with LPS (1 μg/mL) for 6 h followed by ATP (3 mM) for 30 min. **(A–C)** NLRP3 and pro-IL-1β protein levels were indices of inflammasome priming. Representative Western blots are shown in **(A)** and densitometric analyses in **(B,C)**. **(D–F)** Caspase1 activation (p20) and release of mature IL-1β were indices of inflammasome activation. Representative Western blot for caspase1 p20 is shown in **(D)** and densitometric analysis in **(E)**. Protein levels of IL-1β in supernatants were assessed by ELISA. *n* = 3, *p < 0.05 as compared with control; ^#^*p* < 0.05 as compared with LPS+ATP.

**Figure 8:**
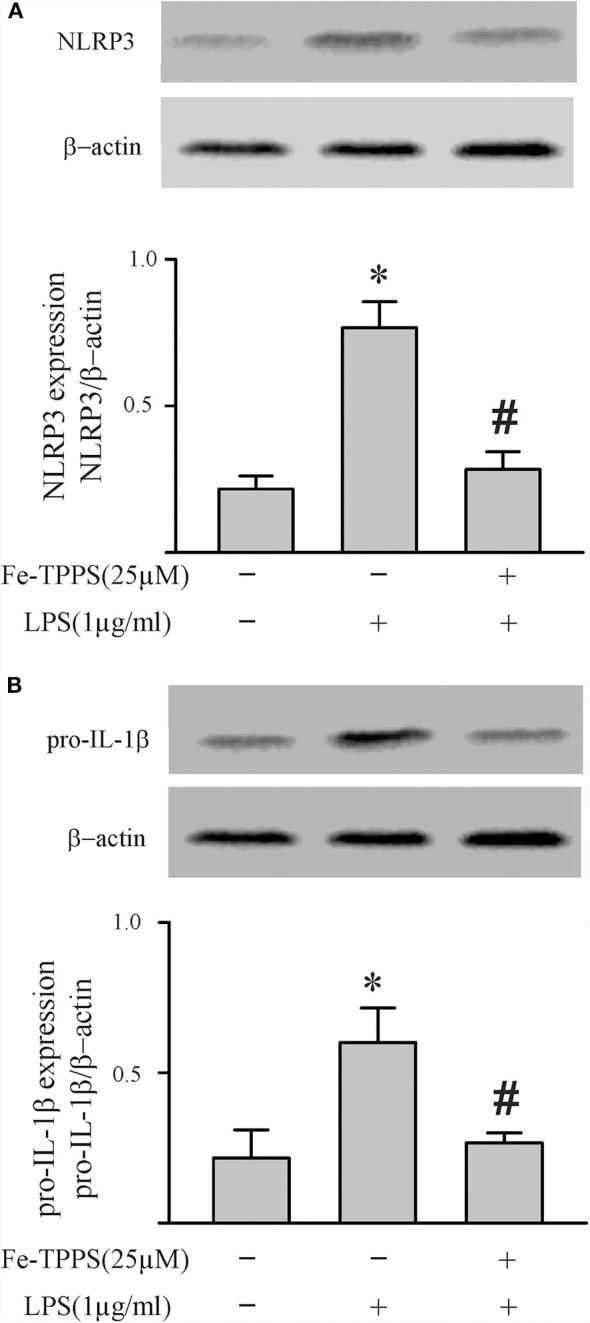
A peroxynitrite decomposition catalyst (Fe-TPPS) decreases LPS-induced priming of the NLRP3 inflammasome. Fibroblasts were pretreated with Fe-TPPS (25 μM) or vehicle for 30 min and subsequently challenged with LPS (1 μg) for 6 h. As indices of inflammasome priming, NLRP3 **(A)** and pro-IL-1β **(B)** protein levels were assessed (Western blot). Representative blots are shown above and densitometric analyses below. *n* = 3, *p < 0.05 as compared with control; ^#^*p* < 0.05 as compared with LPS.

**Figure 9:**
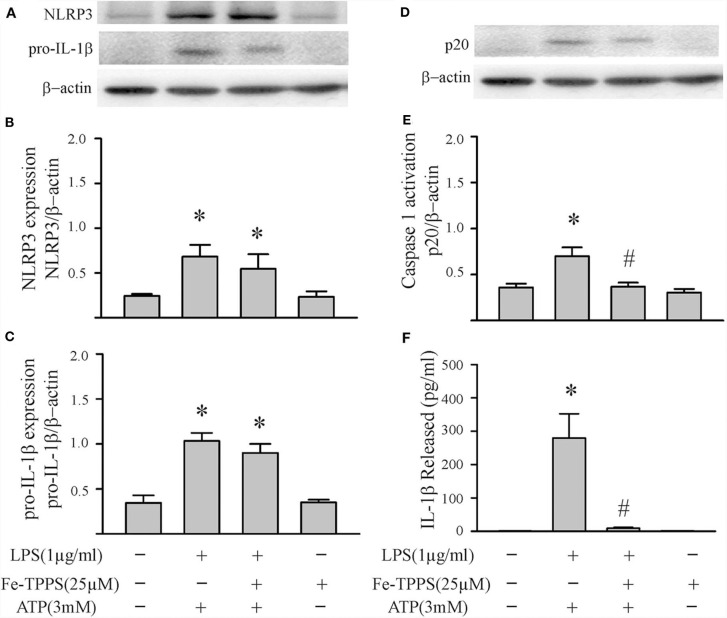
A peroxynitrite decomposition catalyst (Fe-TPPS) decreases ATP-induced activation of the NLRP3 inflammasome. Fibroblasts were challenged with LPS (1 μg/mL) for 6 h. Subsequently, the fibroblasts were treated with Fe-TPPS (25 μM) or vehicle for 15 min, followed by ATP (3 mM) for an additional 30 min. **(A–C)** NLRP3 and pro-IL-1β protein levels were indices of inflammasome priming. Representative Western blots are shown in **(A)** and densitometric analyses in **(B,C)**. Of note, NLRP3 and pro-IL-1β were increased after the initial LPS challenge, but the subsequent challenge with ATP was without effect on these two indices of inflammasome priming. **(D–F)** Caspase1 activation (p20) and IL-1β protein levels in the supernatant were indices of inflammasome activation. Representative Western blot for caspase1 p20 is shown in **(D)** and densitometric analysis in **(E)**. Protein levels of IL-1β were assessed in supernatants by ELISA. *n* = 3, *p < 0.05 as compared with control; ^#^*p* < 0.05 as compared with LPS+ATP.

**Figure 10:**
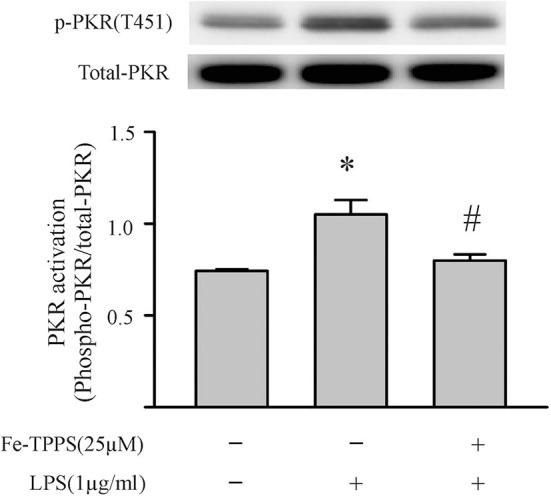
A peroxynitrite decomposition catalyst (Fe-TPPS) prevents the LPS-induced PKR phosphorylation. Fibroblasts were pretreated with Fe-TPPS (25 μM) or vehicle for 30 min and subsequently challenged with LPS (1 μg) for an additional 15 min (see Figure 2 for time course). Thereafter, the fibroblasts were harvested for assessment of PKR phosphorylation on the activation threonine 451 (p-PKR [T451]). Representative Western blot is shown above and densitometric analysis below. *n* = 3, *p < 0.05 as compared with control; ^#^*p* < 0.05 as compared with LPS.

Peroxynitrite is a strong oxidant that is generated by the interaction of superoxide with nitric oxide (22, 46). Previous studies in macrophages have yielded controversial results; peroxynitrite either promotes or inhibits NLRP3 inflammasome activation (18, 19). To assess whether peroxynitrite may be involved in our model, we initially measured nitrotyrosine levels in cardiac fibroblasts. Protein tyrosine nitration yielding 3-nitrotyrosine residues is a commonly used marker of peroxynitrite formed in biological systems (22, 46). As shown in Figure 6, nitrotyrosine levels in cardiac fibroblasts increase in response to LPS challenge, an effect that can be blocked by the peroxynitrite decomposition catalyst, Fe-TPPS. Thus, Fe-TPPS was used to address NLRP3 inflammasome priming/activation. The two-step challenge (LPS followed by ATP) increased fibroblast protein levels of NLRP3, pro-IL-1β, caspase 1 p-20, as well as release of mature IL-1β into the supernatants, effects that were prevented by Fe-TPPS (Figures 7A–E). To specifically address the potential role of peroxynitrite in the priming step, Fe-TPPS was added prior to the sole LPS challenge. The peroxynitrite decomposition catalyst completely abrogated the LPS-induced increases in fibroblast NLRP3 and pro-IL-1β protein levels (Figure 8). To address the role of peroxynitrite in inflammasome activation, Fe-TPPS was added after the LPS priming step but prior to the ATP activation step. Fe-TPPS did not affect NLRP3 or pro-IL-1β protein (Figures 9A,C), but completely blocked the two indices of inflammasome activation, i.e., caspase 1 p20 protein levels in fibroblasts and release of mature IL-1β into the supernatants (Figures 9B,D). Since the PKR inhibitor largely prevented LPS-induced priming of the NLRP3 inflammasome (Figures 5A–C), the role of peroxynitrite in PKR activation was assessed. As shown in Figure 10, Fe-TPPS completely prevented the increase in T451 phosphorylation of PKR.

## Discussion

The functional NLRP3 inflammasome is a multimeric cytoplasmic complex consisting of NLRP3 (sensor), ASC (adaptor), and caspase 1 (effector), a major function of which is to generate IL-1β (7–9). Optimum generation of IL-1β is achieved by a two-step mechanism: an initial priming step followed by an activating signal. In this scenario, a priming signal (e.g., LPS) ensures adequate levels of functional constituents (e.g., NLRP3 and pro-IL-1β) by transcriptional and/or post-transcriptional means. Next, an activating signal (e.g., ATP) promotes assembly of the tripartite inflammasome complex, oligomerization of NLRP3 with ASC, which subsequently recruits pro-caspase1. Upon assembly, autocleavage of pro-caspase1 activates the enzyme, which, in turn, generates the active form of IL-1β from the latent pro-IL-1β. The NLRP3 inflammasome has been extensively characterized in monocytes/macrophages, resulting in a bewildering array of DAMPs and PAMPs that can serve as primers and/or activators (7). With respect to signaling pathways, both oxidative stress and phosphorylation events have been invoked in both priming and activation steps of the NLRP3 inflammasome (6–8, 15, 35). The results of the present study are in accord with this view. Herein, we provide evidence to support a role for oxidants (peroxynitrite) and phosphorylation (protein kinase R) in the regulation of both priming and activation of the NLRP3 inflammasome.

A growing body of evidence indicates that cardiac non-immune cells, specifically myocytes and fibroblasts, can also assemble and activate the NLRP3 inflammasome during the development of various cardiomyopathies. In experimental models of cardiac infarction (DAMP-mediated) or endotoxemia (PAMP-mediated), activation of the NLRP3 inflammasome in either cardiac fibroblasts (36–39, 47) or myocytes (47–49) may contribute to the resultant pathology, cardiac fibroblasts being considered as the more important of the two (36–38). On the other hand, the upregulation of cardiomyocyte NLRP3 inflammasome has been implicated as causative in the inflammatory component of the cardiomyopathy resulting from pacing-induced arrhythmogenesis (11) or pressure overload (50). While the mechanisms involved in priming/activation of the inflammasome in the arrhythmogenic model are not clear, the cardiac failure induced by pressure overload was dependent on NLRP3 inflammasome activation by the protein kinase, CaMKIIδ. Further, since the cardiomyocytes were not injured during development of cardiomyopathy in the pressure-overload model, a role for DAMPs or PAMPs was negated (50). Collectively, these observations indicate that the relative importance of these two resident cell populations to the inflammatory response of the heart is most likely context-dependent and the mechanisms/pathways involved in inflammasome activation may differ.

In the present study, we addressed potential mechanisms involved in the priming and activation of the NLRP3 inflammasome in cardiac fibroblasts using the two-step model, i.e., LPS priming and ATP activation (7–10, 12). The rationale for this approach was two-fold. First, our previous studies indicate that the NLRP3 inflammasome of cardiac fibroblasts generates IL-1β that subsequently depresses the contractile activity of adjacent cardiomyocytes (38, 39). Second, the sequential challenge of fibroblasts with LPS (a PAMP) and ATP (a DAMP) mimics a scenario of potential relevance to endotoxemia. To address inflammasome priming, we focused on induction of NLRP3 and pro-IL-1β, while indices of inflammasome activation included activation of caspase1 as well as release of mature IL-1β. These readouts are in accord with LPS-induced priming and ATP-induced activation of the NLRP3 inflammasome in myeloid cells (7, 8, 11), fibroblasts (38, 39, 51), and cardiomyocytes (49).

Based on our initial feasibility studies (Figure 1), we targeted protein kinase R (PKR) as a potential upstream mediator of NLRP3 inflammasome priming/activation. Both a PKR inhibitor and PKR siRNA blunted the LPS-induced increases in fibroblast NLRP3 and pro-IL-1β (Figures 4, 5). Further, in LPS-primed fibroblasts, the ATP-induced activation of caspase1 and secretion of mature IL-1β were reduced by the PKR inhibitor (Figures 5D–F). A role for PKR in the regulation of the NLRP3 inflammasome in macrophages is controversial. PKR can enhance (28–30, 41, 52), suppress (32), or have no effect (33) on the activation of LPS-primed inflammasome. Even at the level of priming, i.e., LPS-TLR4-NFκB signaling, PKR activation was either required (43, 44, 47) or inconsequential (53). While there is no satisfactory explanation for these diverse findings in myeloid cells, proposed contributing factors include different macrophage populations assayed, priming/activating signals, as well as approaches to ablate PKR function (28, 32, 33, 52, 54). Irrespective, the bulk of the evidence supports a role for PKR in either priming or activation of the NLRP3 inflammasome (54). Herein, using a potent inhibitor of PRK kinase activity or siRNA targeting PKR, we provide evidence indicating that PKR promotes both the priming as well as the activation of the NLRP3 inflammasome in cardiac fibroblasts (Figures 2–5).

Another controversial issue addressed in the present study is the role of reactive oxygen species (ROS) and nitrogen species (RNS) as well as downstream peroxynitrite in the priming and activation of the NLRP3 inflammasome. ROS have been proposed as a potential final common pathway by which different priming and activating signals converge to enhance the functional activity of the NLRP3 inflammasome (7, 8, 12, 13, 15). ROS have been implicated in both the LPS-TLR4 priming (14) and the ATP-P2X7R activating (18) steps. The cellular sources of ROS are mitochondria and/or NADPH oxidase (NOX), both of which generate superoxide (7, 12, 13). NO production by iNOS in macrophages is increased by LPS (55, 56), an effect enhanced by ATP (57). Of relevance herein, the anti-bacterial function of macrophages has been attributed to peroxynitrite generated by interaction of NOX-derived superoxide and iNOS-derived NO (20, 58, 59). The results of the present study support a role for peroxynitrite in both the priming and activating steps of the NLRP3 inflammasome in cardiac fibroblasts (Figures 6–9).

Peroxynitrite, or its downstream products, has been implicated in redox signaling (22, 60). The potential signaling function of peroxynitrite, coupled to the results of the present study in which limiting either peroxynitrite or PKR activity nullified both the LPS-priming and ATP-activation steps of the inflammasome, prompted us to assess the role of peroxynitrite in PKR activation. As shown in Figure 10, the LPS-induced phosphorylation of PKR was prevented by preincubation of the fibroblasts with the peroxynitrite decomposition catalyst. Peroxynitrite can activate kinases (e.g., IκB kinase), or alter protein structure (cysteine oxidation), or both (22, 60). Of interest, peroxynitrite can activate the kinase, CaMKIIδ (61), which has been implicated in NLRP3 inflammasome activation in cardiomyocytes (50). Further studies are warranted to address the specific mechanisms by which peroxynitrite modulates PKR phosphorylation.

As a caveat, a potential limitation of the present study is that the cardiac fibroblast cultures used were only 95% pure. The isolation procedure selects against cardiac myocytes as well as endothelial cells and, thus, the most likely cells in 5% contaminating population were smooth muscle cells. While we cannot completely exclude the contribution of any contaminating cells in our fibroblast cultures, their impact on the results obtained and interpretation thereof would most likely be negligible.

**Figure 11:**
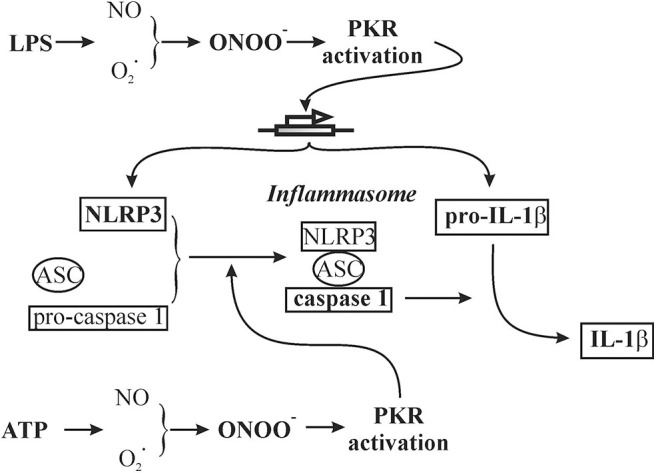
Schematic of a working hypothesis of a role for the peroxynitrite/protein kinase R pathway in both LPS priming and subsequent ATP activation of the NLRP3 inflammasome in cardiac fibroblasts. The specific components addressed in the present study are in bold.

In summary, NLRP3 inflammasome function has been extensively studied in macrophages; molecular mechanisms involved in inflammasome function are not entirely clear and often contradictory. The present study is the first to address two controversial issues regarding the regulation of NLRP3 inflammasome function in non-immune cells, i.e., cardiac fibroblasts. Specifically, we addressed the roles of protein kinase R and peroxynitrite. The results obtained support a role for a peroxynitrite/protein kinase R pathway in the triggering of the NLRP3 inflammasome in cardiac fibroblasts (Figure 11). The approaches used to prime (LPS) and activate (ATP) the fibroblast inflammasome are relevant to the role of fibroblasts in the cardiac inflammatory response of sepsis/endotoxemia.

## Data Availability Statement

The raw data supporting the conclusions of this article will be made available by the authors, without undue reservation.

## Ethics Statement

The animal study was reviewed and approved by University of Western Ontario.

## Author Contributions

TL, AT, and XX performed experiments and data analysis. TR and PK wrote the manuscript. All authors contributed to the article and approved the submitted version.

## Conflict of Interest

The authors declare that the research was conducted in the absence of any commercial or financial relationships that could be construed as a potential conflict of interest.
