# Supplementary figures and images for: Peroxynitrite/PKR Axis Modulates the NLRP3 Inflammasome of Cardiac Fibroblasts
Source: Front Immunol. 2020 Sep 25;11:558712. doi: 10.3389/fimmu.2020.558712 (PMC7545724; doi:10.3389/fimmu.2020.558712)

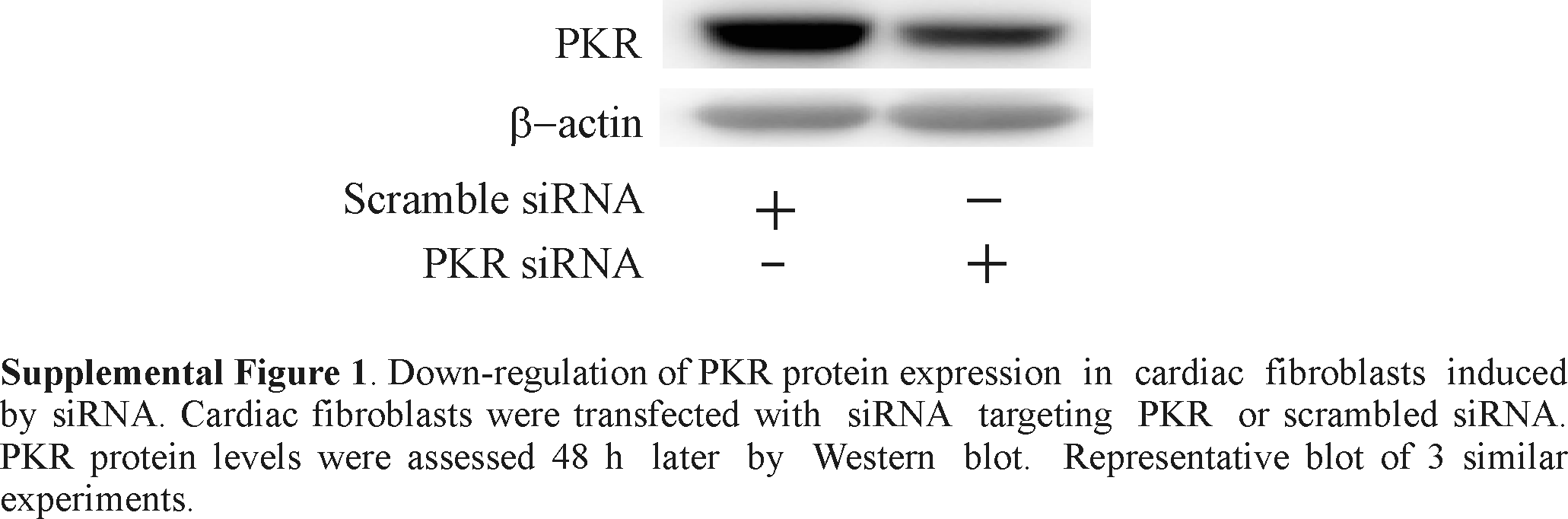

Supplement: Supplementary file 1 [file Image_1.TIF]
